# MCC Regulator of WNT Signaling Pathway (MCC) Is a Podocyte Essential Gene

**DOI:** 10.3389/fmed.2021.777563

**Published:** 2021-12-02

**Authors:** Hui Song, Lulu Zhuang, Xiaodong Xu, Jingsong Shi, Weixin Hu, Zhihong Liu, Shaolin Shi

**Affiliations:** National Clinical Research Center for Kidney Diseases, Affiliated Jinling Hospital, Medical School of Nanjing University, Nanjing, China

**Keywords:** MCC, podocyte, podocytopathy, lamellipodia, RNA-seq

## Abstract

Podocytes are an integral part of the glomerular filtration barrier. Many genes are already known to be essential for podocyte survival, structure and function, but there are more podocyte essential genes to be identified. By single-cell RNA-seq of mouse podocytes, we detected the expression of gene encoding MCC regulator of WNT signaling pathway (MCC) in majority of the podocytes and speculated that MCC is essential for podocytes. We confirmed MCC expression in mouse podocytes and further showed its expression in human podocytes. To experimentally prove the essentiality of MCC for podocytes, we knocked down MCC in cultured podocytes and found marked morphological change of cell shape, cytoskeletal F-actin stress fiber disruption, increased apoptosis, and downregulation of podocyte essential genes, CD2AP and WT1, demonstrating that MCC is essential for podocytes. Since MCC has been implicated in cell cycle and β-catenin signaling, we examined the expression of cell cycle related genes and activity of β-catenin in the MCC knockdown podocytes, but did not find significant changes. To further explore the mechanism underlying the role of MCC in podocytes, we performed RNA-sequencing and bioinformatics analysis of MCC knockdown podocytes and found a significant enrichment of the regulated genes in lamellipodia formation. Consistently, we found that MCC is present in lamellipodia and MCC knockdown resulted in loss of lamellipodia in the cells. Lastly, we found that MCC was downregulated in podocytes treated with puromycin aminonucleosides and in glomeruli of diabetic mice and FSGS patients, implicating MCC is involved in the development of podocytopathy and proteinuria. In conclusion, MCC is potentially essential for podocytes and its downregulation may be involved in podocytopathy.

## Introduction

Podocytes attach to glomerular base membrane (GBM) to cover the capillaries. They have extensive foot processes which are interdigitally arranged to form slit diaphragms, allowing small molecules, e.g., salts and glucose, to pass through, while retaining the macromolecules, e.g., albumin in the blood vessels (1, 2). Therefore, podocytes are an integral part of the glomerular filtration barrier. Podocyte injury underlies and initiates focal segmental glomerulosclerosis (FSGS) (3). Podocyte injury is also involved in many other types of glomerular diseases, e.g., membranous diseases, diabetic nephropathy, IgA nephropathy, etc. (4). It is essential to understand podocyte biology and pathogenesis.

Podocytes are also characterized by terminal differentiation and do not proliferate life-time (5, 6). Podocytes express a set of CDK inhibitors that may help maintain cellular quiescence and differentiation state, e.g., p21Cip1, p27Kip1, and p57Kip2 (7, 8). In many types of injury and disease, podocytes exhibit expression change of the cell cycle proteins, including the CDK inhibitors and cyclin molecules. The downregulation of CDK inhibitors could induce podocytes to re-enter cell cycle for proliferation (9); however, due to the intrinsic barrier to normal cell cycle in differentiated podocytes, the podocytes cannot complete mitosis, and the mitotic arrest results in mitotic catastrophe that leads to cell death (10, 11). On the other hand, the upregulation of CDK inhibitors can cause cell cycle arrest in podocytes in certain diseases, e.g., diabetic nephropathy, leading to hypertrophy and cell death (12–15). Therefore, it is essential for podocytes to possess a machinery that optimally maintains their differentiation and quiescence. However, the machinery has not been completely understood.

MCC regulator of WNT signaling pathway (MCC) is a potential colorectal tumor suppressor gene and may negatively regulate cell cycle progression. The gene encodes a phosphoprotein that is associated with the plasma membrane and membrane organelles. It has been shown that MCC overexpression can inhibit the entry into S phase in cell cycle (16). As a tumor suppressor, MCC inhibits Wnt/β-catenin signal transduction (17, 18). Interestingly, MCC was found to act as an oncogene in B cells and its knockdown induced apoptosis in human multiple myeloma (19).

We previously performed single-cell RNA-seq analysis of mouse podocytes and found a huge heterogeneity of gene expression between individual podocytes with a correlation coefficient of only 0.2. There were only a small proportion of genes that were commonly expressed in all individual podocytes (20). We speculated that commonly-expressed genes are indispensable for podocyte survival and structural and functional homeostasis; while the differentially expressed genes could be dispensable. We identified 335 genes that were expressed in all individual podocytes sequenced and demonstrated the essentiality of some genes for podocytes (20). In addition, we identified genes whose expression was detected in most of the podocytes sequenced. We speculated that these genes were also expressed in all the podocytes but the expression detection might have failed in some cells due to technical variations in the single-cell RNA-seq process; thus, they could be potential podocyte essential genes as well.

In the present study, we explored the list of genes expressed in majority of the sequenced podocytes and focused on MCC. This was because MCC expression was detected in 18 out of the 20 sequenced podocytes and, as described above, it regulates cell cycle and Wnt/β-catenin signaling, whose abnormality is known to be involved in podocyte injury. We knocked down MCC in cultured podocytes, followed by characterization of cell injury and RNA-seq analyses. The results showed that MCC deficiency induced podocyte injury. However, the injury did not involve deregulation of cell cycle and Wnt/β-catenin, unexpectedly, but instead loss of lamellipodia. This study has thus identified MCC as a potential podocyte essential gene.

## Materials and Methods

### Database Mining and Bioinformatics Analysis

We extracted information of interest from following databases: data of MCC expression were obtained from the single podocyte sequencing (GSE88814) and bulk sequencing of mix mesangial and endothelial cells (GSE89263). MCC expression in mouse and human podocytes were also available in KIT database (http://humphreyslab.com/SingleCell/). We used the Human Protein Atlas (www.proteinatlas.org) to examine MCC protein expression in human glomeruli. To investigate MCC expression changes in diseases, we searched Nephroseq (www.nephroseq.org).

### Reagents

The antibodies against MCC, CD2AP, total β-catenin, p16, p21, p27, p57, CDK1, Cyclin E1, Cyclin D2, PCNA, and GAPDH (Proteintech, USA); antibodies against active β-catenin, total ERK1/2 and phospho-ERK1/2, total AKT and phospho-AKT (Cell Signaling Technologies, USA); WT1 antibody (Abcam, USA); puromycin aminonucleosides (Sigma, USA); Alexa Fluor® 647 Annexin V and Propidium Iodide (Biolegend); Reverse transcription kit, DRR037A (Takara); quantitative PCR kit (Thermo Fisher Scientific); RIPA cell lysis buffer, BCA protein quantification kit (Beyotime, Shanghai); RNA extraction kit (Takara).

### Culture of Immortalized Human Podocytes

The human podocyte cell line was provided by Dr. Saleem M (University of Bristol, United Kingdom). The cells were cultured in RPMI 1640 containing 10% fetal bovine serum and pennicilin/streptomycin (100 U/ml of each) (Gibco, USA) and 1% insulin-transferrin-selenium (ITS, Invitrogen). Podocytes were grown at 33°C and switched to 37°C, followed by incubation for ~10 days for differentiation.

### SiRNA and PAN Treatment of Podocytes

The siRNA of MCC was synthesized by HanBio (Shanghai) according to the sequence, 5′-CAUCACUAAAGGGAGAUAU (sense strand). Podocytes were cultured at 37°C for differentiation in 6- or 12-well plates. Transfection of the siRNAs into the differentiated cells was performed using the Lipofectamine® RNAiMAX reagent (Invitrogen, CA) according to the instruction manual. The medium was changed after 6 h, and the cells were harvested 36 or 72 h after transfection. We used 50 or 100 μg/ml PAN (Sigma-Aldrich, USA) to treat podocytes followed by RNA and cell lysate preparation.

### Immunofluorescence Staining

We followed the method described previously (21).

### Phalloidin Staining of F-Actin

F-actin was stained using rhodamine-labeled phalloidin, and the resulting microscopy images were digitized. The images were converted to 8-bit and then inverted. Rhodamine-stained areas were quantified using ImageJ software (National Institutes of Health, Bethesda, MD). Mean actin per pixel and total actin content per cell were calculated and given as arbitrary units as previously described (21).

### Quantification of the Actin Cytoskeleton

Rhodamine-labeled phalloidin was used to stain F-actin in the podocytes, and the resulting images were obtained by confocal microscopy and digitized. The rhodamine-stained areas of the actin fibers were converted to black pixels and then inverted, followed by quantification using ImageJ software (NIH). The grayscale values ranged from 0 (black) to 200 (white, the maximal actin content). The mean podocyte actin content per pixel and the total actin content per cell were calculated and expressed as AU.

### RNA Extraction and qPCR Analysis

Cultured podocytes were subjected to total RNA extraction using the RNA Extraction Kit (Takara). Reverse transcription was performed using the kit from Takara (RR036A). The primers were: MCC, 5'-ACTCACTTCAGGACTGCTCCA-3′ (forward) and 5′-ATTCAGCCGTTCTGTTTCCAC-3′ (reverse); CD2AP, 5′-AAAAGCCCTTAATCCTACAGT-3′ (forward) and 5′-CCTTCTTTACCATTAAGTTCGC-3′ (reverse); WT1, 5′-AAGCAGCTAACAATGTCTGGT-3′ (forward) and 5′-TTCCATCCCCAGCGAAAACGA-3′ (reverse); p15, 5′-ACCCTGCCACTCTCACCCGAC-3′ (forward) and 5′-CCCAGGCATCGCGCACGTCCA-3′ (reverse); p16, 5′-GGGTTTTCGTGGTTCACATCC-3′ (forward) and 5′-CTAGACGCTGGCTCCTCAGTA-3′ (reverse); p21, 5′-AGGTGGACCTGGAGACTCTCAG-3′ (forward) and 5′-TCCTCTTGGAGAAGATCAGCCG-3′ (reverse); p27, 5′-CGGAGCACCCCAAGCCCTC-3′ (forward) and 5′-CAAGCTGCCCTTCTCCACCT-3′ (reverse); p57, 5′-CAAAGCCCAAAGAGCCCCGAG-3′ (forward) and 5′-CTGCTACATGAACGGTCCCAG-3′ (reverse); PCNA, 5′-ATTTGCACGTATATGCCGAGA-3′ (forward) and 5′-GCAGAAAATTTCACTCCGTCT-3′ (reverse); CDK1, 5′-GGAAACCAGGAAGCCTAGCATC3′ (forward) and 5′-GGATGATTCAGTGCCATTTTGCC-3′ (reverse); CDK4, 5′-CCATCAGCACAGTTCGTGAGGT-3′ (forward) and 5′-TCAGTTCGGGATGTGGCACAGA-3′ (reverse); Cyclin B1, 5′-GACCTGTGTCAGGCTTTCTCTG-3′ (forward) and 5′-GGTATTTTGGTCTGACTGCTTGC-3′ (reverse); Cyclin D2, 5′-GAGAAGCTGTCTCTGATCCGCA-3′ (forward) and 5′-CTTCCAGTTGCGATCATCGACG-3′ (reverse); Cyclin E1, 5′-TGTGTCCTGGATGTTGACTGCC-3′ (forward) and 5′-CTCTATGTCGCACCACTGATACC-3′ (reverse); Cyclin E2, 5′-CTTACGTCACTGATGGTGCTTGC-3′ (forward) and 5′- CTTGGAGAAAGAGATTTAGCCAGG-3′ (reverse); 18s rRNA, 5′-TTCTCGATTCCGTGGGTGG-3′ (forward) and 5′-AGCATGCCAGAGTCTCGTTC-3′ (reverse). SYBR Green dye was used in the qPCR. The thermal condition was 95°C/30 s for denature, followed by 40 cycles of 95°C/5 s−60°C/30 s on the ABI 7900HT Fast Real time System. Threshold cycle (CT) values were determined and the relative abundance of the mRNA was calculated with the formula 2^−ΔΔCt^.

### Western Blot Analysis

Podocytes were washed with cold PBS and then lysed with 150 μl of RIPA buffer containing proteinase inhibitors cocktail (Roche) and phosphatase inhibitors. The lysates were incubated on ice and then centrifuged 12,000 g for 15 min at 4°C. The supernatants were transferred to fresh tubes and then subjected to protein concentration measurement with BCA protein kit (Bio-Rad). After mixed with loading buffer, the samples were boiled at 98°C for 5 min. 10 or 8% SDS-PAGE was used to fractionate the samples, and semi-dry transfer system (Bio-rad) was used to transfer the protein from the gel to PVDF membrane. The blot was blocked with 5% milk in TBST solution (20 mM Tris-HCl, PH 7.14, 150 mM NaCl, 0.1% Tween-20) for 60 min at room temperature, and then incubated with an antibody overnight at 4°C. After washed with TBST for 3 times, the blot was incubated with HRP-labeled secondary antibody for 1 h at room temperature. After washed, ECL system (Millipore) was used to detect the protein. Cell lysates were prepared using radioimmunoprecipitation assay (RIPA) buffer containing a protease inhibitor cocktail (Roche) and a phosphatase inhibitor. The blot was incubated with a primary antibody of interest.

### Flow Cytometric Analysis of Apoptosis via Annexin V Staining

After treatment, the podocytes were collected, washed twice with ice-cold PBS, resuspended in 200 μl binding buffer, and then incubated with FITC-conjugated annexin V at a final concentration of 0.5 μg/ml at room temperature for 15 min. Then, the cells were washed, centrifuged, and resuspended in 500 μl binding buffer. The cells were stained with 50 μg/ml propidium iodide at room temperature for 5 min, followed by flow cytometric analysis using a FACScan flow cytometer and CellQuest software (BD).

### RNA-Sequencing

Total RNA was extracted from the cultured podocytes using Trizol (Invitrogen, USA) following the manual instruction. Paired-end libraries were made using the TruSeq® RNA Sample Preparation Kit (Illumina, USA) following the Sample Preparation Guide of the kit. Briefly, the poly-A containing mRNA molecules were incubated with oligo poly-T magnetic beads, followed by washing and elution to obtain purified mRNA molecules for library construction. Purified libraries were quantified by Qubit® 2.0Fluorometer (Life Technologies, USA) and examined with the Agilent 2100 bioanalyzer (Agilent Technologies, USA) to validate the insert size and measure the concentrations. Clusters were generated by cBot with the library diluted to 10 pM and then sequenced on the Illumina HiSeq X-ten (Illumina, USA). The library construction and sequencing were performed at Shanghai Biotechnology Corporation (China).

### Data Analysis for Gene Expression

We preprocessed the sequencing raw reads by removing the reads of rRNA, sequencing adapters, short-fragments, as well as other low-quality reads. We used Hisat2 (version:2.0.4) (22) to map the cleaned reads to the human GRCh38 reference genome with two mismatches. After genome mapping, we used Stringtie (version:1.3.0) (23, 24) with a reference annotation to generate FPKM values for known gene models. Differentially expressed genes were determined using the edgeR (25). The p-values of significance in multiple tests were determined by the false discovery rate (FDR) (26, 27). The fold-changes were calculated based on the FPKM in each sample. The differentially expressed genes were determined by FDR ≤ 0.05 and fold-change ≥1.5.

### Differential Expression Analysis

Differential expression analysis was performed with R package “DESeq2”[@LoveDESeq2]. The estimation steps were wrapped into a single function “DESeq”. Results tables were generated with log2 fold changes, *p* values and adjusted *p* values. Finally, log fold change shrinkage was used to improve on the previous estimator.

### Statistics

Data are presented as the mean ± SD. Differences between 2 groups were analyzed using a 2-tailed Student's *t* test and incorporated into GraphPad Prism 5 software (GraphPad Software). *P* < 0.05 was considered statistically significant.

## Results

### MCC is Expressed in Podocytes

**Figure 1:**
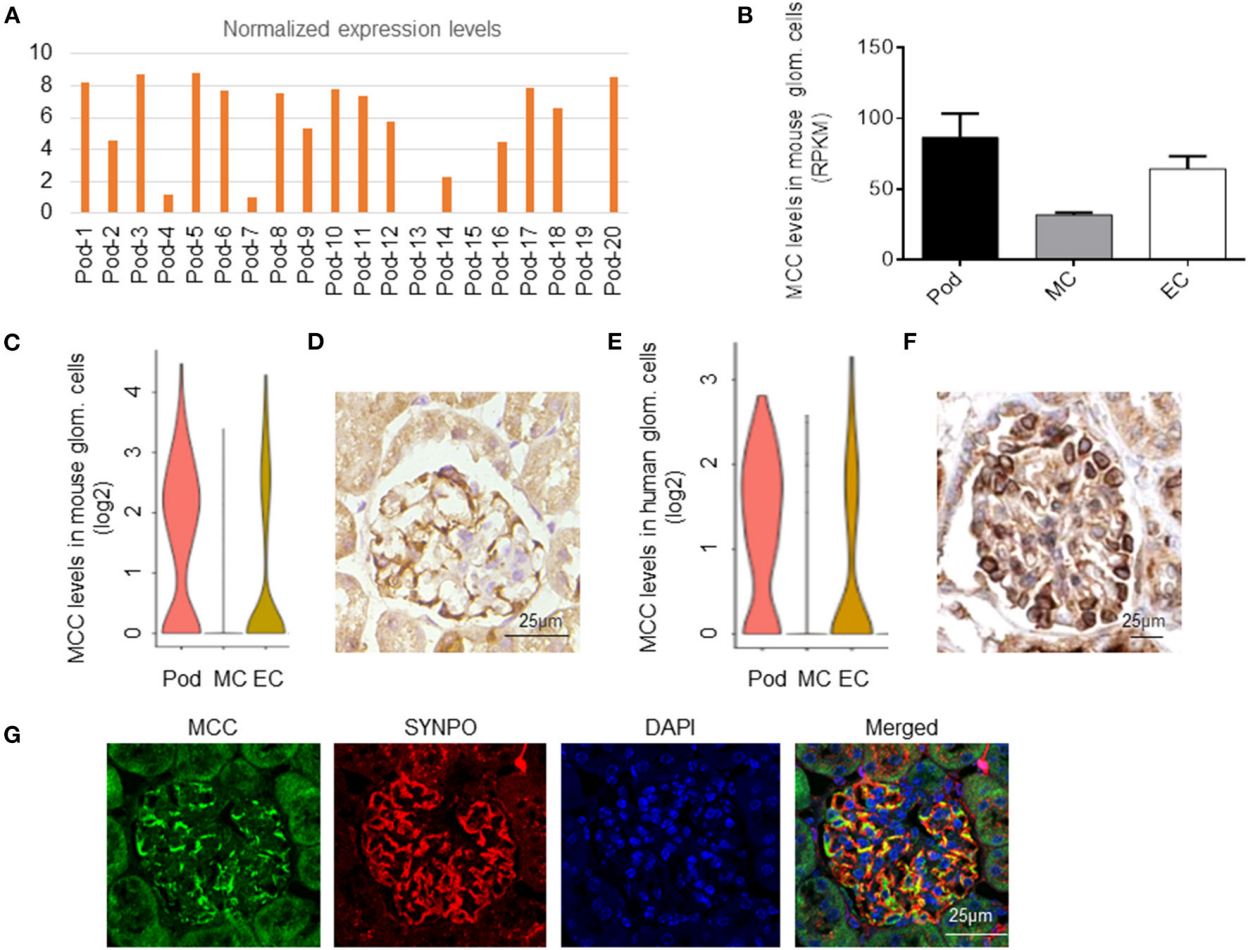
MCC expression in podocytes in mouse and human. **(A)** MCC mRNA was detected in 18 out the 20 mouse podocytes subjected to single-cell RNA-seq analysis (GEO: GSE88814). **(B)** MCC expression in mouse podocytes, mesangial cells and glomerular endothelial cells from the bulk sequencing of mouse glomerular cells (GEO: GSE123179). **(C)** MCC expression in glomerular cell types according to the database, KIT (http://humphreyslab.com/SingleCell/). **(D)** Immunohistochemical staining of MCC in a mouse glomerulus, showing a staining pattern characteristic of podocytes. **(E)** MCC expression in human glomerular cell types, podocytes (Pod), mesangial cells (MC) and endothelial cells (EC) according to the KIT database above. **(F)** The immunohistochemical staining of MCC from the Human Protein Atlas (www.proteinatlas.org) shows that MCC expression is relatively specific to podocytes. **(G)** Fluorescence co-immunostaining of MCC and SYNPO in mouse kidney, showing the localization of MCC in foot processes of podocytes.

We found that MCC expression was detected in 18 out of the 20 podocytes sequenced and > 5 fold in podocytes versus mesangial and endothelial cells [Figure 1A, (20)]. We also analyzed the bulk RNA-seq data of purified mouse podocytes, mesangial and endothelial cells (GEO database: GSE123179) (28), respectively, and confirmed the expression of MCC in mouse podocytes, which was higher than that in mesangial and endothelial cells (Figure 1B). We further confirmed MCC expression in podocytes in the single-cell RNA-seq database, KIT (Kidney Interactive Transcriptomics) (http://humphreyslab.com/SingleCell/) (Figure 1C). Furthermore, we performed immunohistochemical staining of MCC in mouse kidney and found expression of MCC in podocytes (Figure 1D). As gene expression conservation across species in a cell type reflects essentiality of the gene for the cell type, we investigated MCC expression in human podocytes. We found in above KIT database that MCC is expressed in human podocytes at a level higher than that in mesangial and endothelial cells (Figure 1E). Consistently, from the Human Protein Atlas (www.proteinatlas.org), MCC was found mainly expressed in podocytes as shown by the immunohistochemical staining pattern characteristic of podocytes along the periphery of glomerular tuft (Figure 1F). These results together demonstrate that MCC is expressed relatively specifically in podocytes in glomeruli in an evolutionarily conserved manner.

### MCC is Localized in Cytoplasm, Nucleus, and Lamellipodia of Podocytes

**Figure 2:**
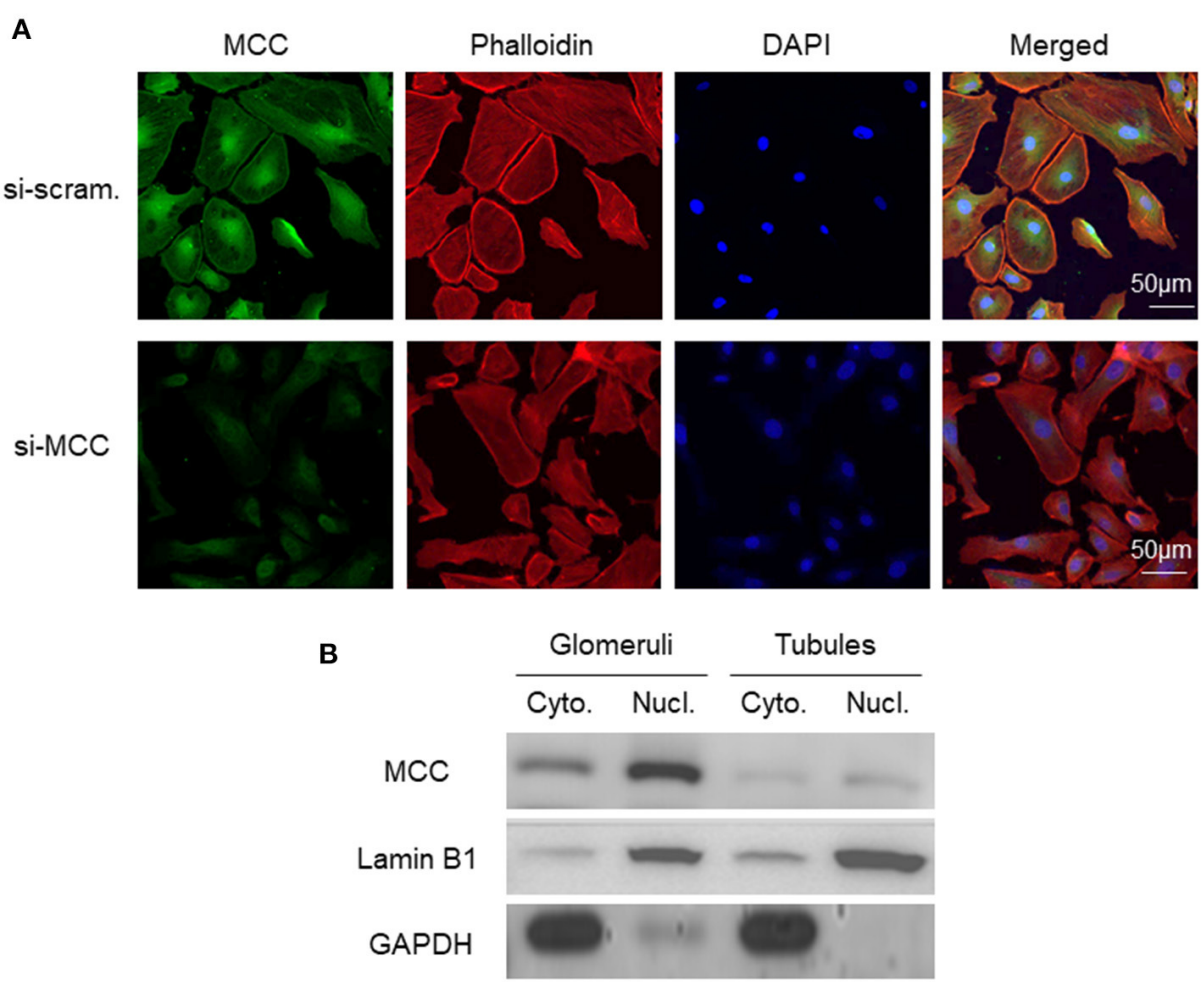
Subcellular localization of MCC protein in podocytes in culture. **(A)** Fluorescence immunostaining of MCC and DAPI in cultured podocytes treated with scramble and si-MCC, respectively, which shows MCC protein in the cytoplasm, nucleus and periphery of the cells. **(B)** Representative immunoblotting of MCC in the cytoplasmic and nuclear fractions of human glomerular cells. GAPDH served the cytoplasmic marker, while Lamin B1 the nuclear marker. MCC expression in tubules was also examined, showing cytoplasmic and nuclear distribution; however, the protein levels were both significantly lower than that in glomerular cell cytoplasm and nuclei, respectively.

To determine the subcellular localization of MCC protein in podocytes, we performed fluorescence immunostaining of MCC and DAPI in cultured podocytes. The result showed that MCC protein was present in the cytoplasm and nucleus, as well as the periphery of the cells (Figure 2A). The treatment using siRNA of MCC (si-MCC) resulted in a marked decrease of intensity in these areas, demonstrating the specificity of staining. To further prove the cytoplasmic and nuclear localization of MCC in podocytes, we separated cytoplasmic and nuclear fractions of glomerular cells, and performed immunoblotting of MCC with cytoplasmic marker (GAPDH) and nucleic marker (Lamin B1). The result clearly showed MCC protein in both cytoplasmic and nucleic samples of glomerular cells (Figure 2B), confirming that MCC protein localizes in both cytoplasm and nucleus of podocytes.

### MCC Deficiency Caused Podocyte Injury

**Figure 3:**
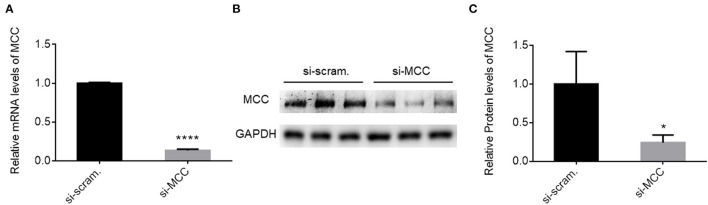
MCC siRNA treatment resulted in marked reduction of MCC expression. **(A)** qPCR analysis of MCC mRNA in si-scramble control and si-MCC cells which were harvested for mRNA preparation 24 h post transfection. **(B)** Immunoblotting of the MCC protein in the cells in A. **(C)** Quantification of the MCC protein levels in the blot in B. Each column represents the mean ± SD of the triplicate samples. ^*^p < 0.05, statistically significant. ^****^p < 0.0001, extremely significant.

To prove the essentiality of MCC for podocytes directly, we knocked down MCC by siRNA (si-MCC) in cultured podocytes. We tested four independent siRNAs against MCC, and they all showed excellent efficiency in MCC mRNA elimination at 24 h post si-MCC transfection. Figure 3A shows the knockdown efficiency for one of them by qPCR. Consistently, the protein level of MCC was accordingly reduced in the cells (Figures 3B,C). We then performed various experiments to examine podocyte changes in the absence of MCC.

**Figure 4:**
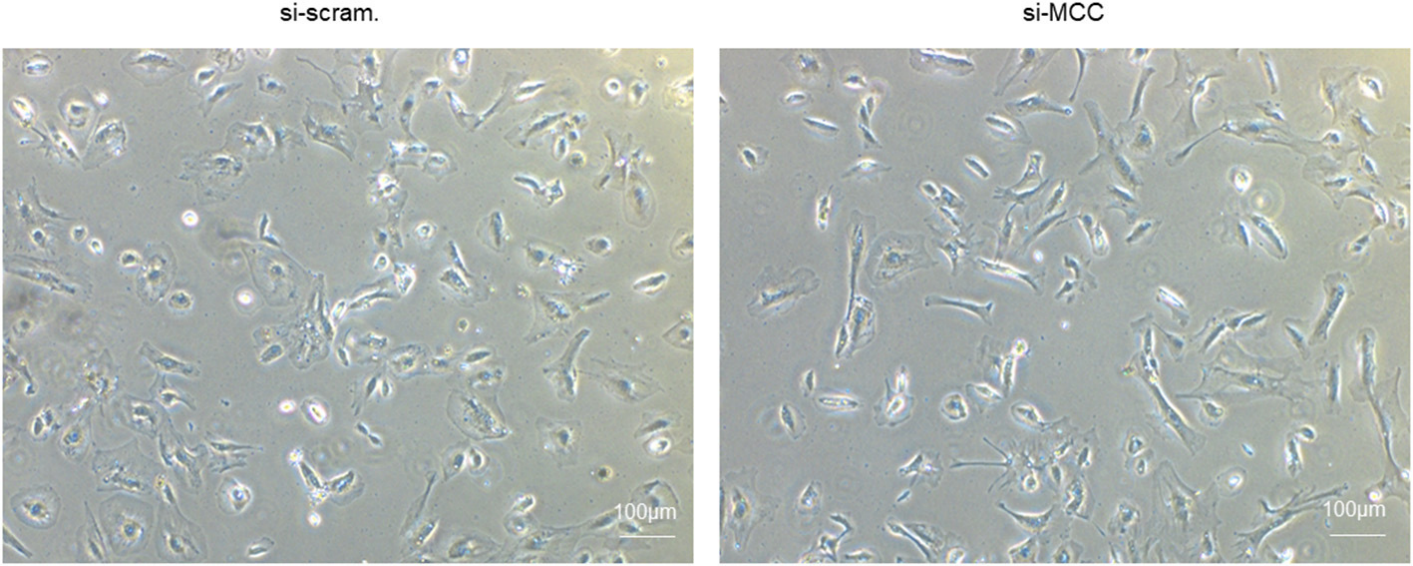
Morphological change of podocytes treated with si-MCC. At 72 h post transfection of si-MCC, the podocytes exhibited an elongated shape compared with the scramble control cells.

**Figure 5:**
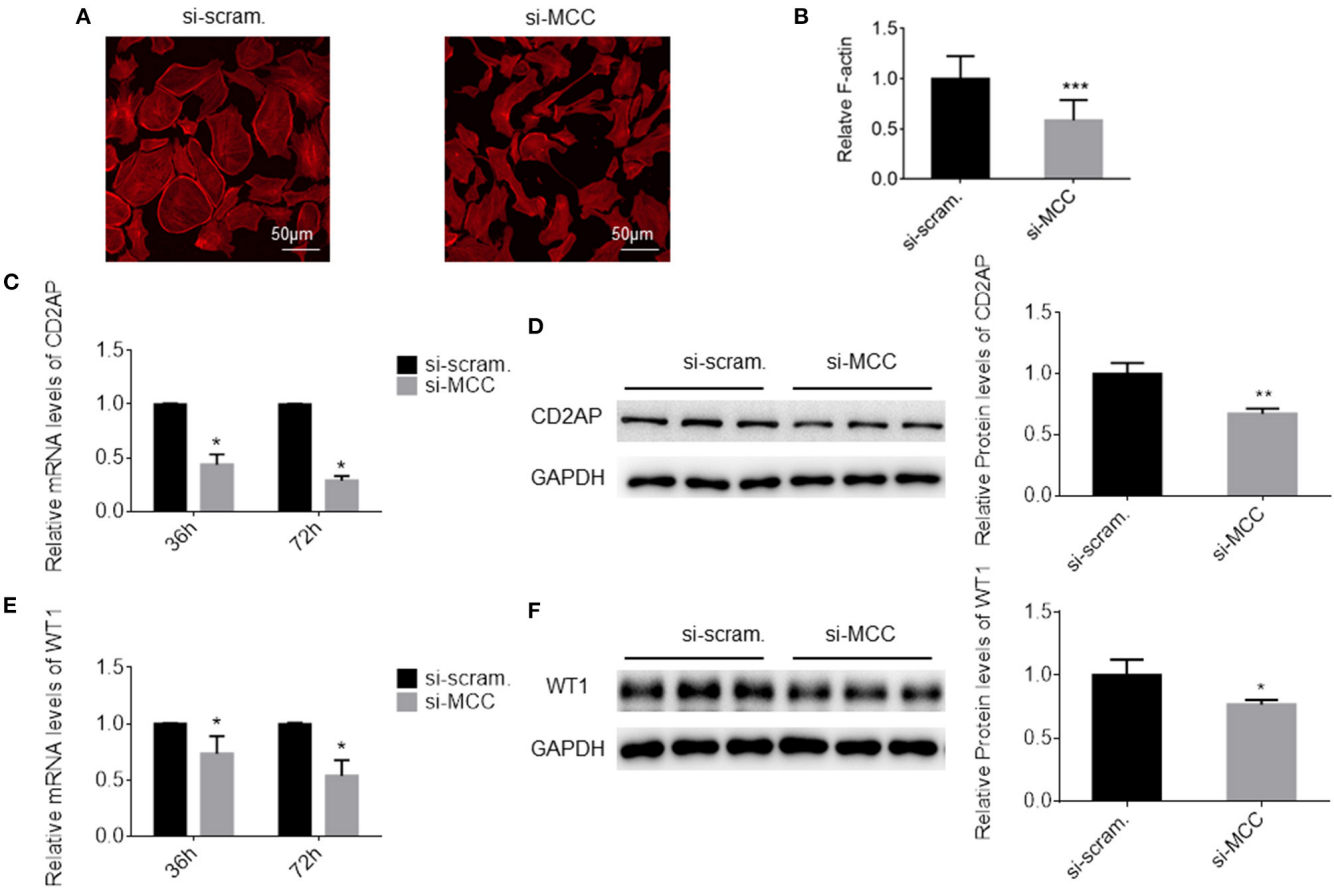
MCC silencing resulted in injury in podocytes. **(A)** Phalloidin staining of the cells treated with scramble control and si-MCC for 72 h. **(B)** Quantification of the staining intensity using ImageJ showed a significant reduction of actin stress fibers in the cells treated with si-MCC compared with scramble control. **(C)** qPCR quantification of CD2AP mRNA in control cells and the cells treated with si-MCC at 36 h and 72 post transfection, respectively. **(D)** Immunoblotting of the CD2AP protein in the cell samples in C to confirm CD2AP downregulation at the protein level. Quantification of the blot is shown on the right. **(E)** qPCR analysis of WT1 in the cells treated with scramble control and si-MCC, respectively. **(F)** Immunoblotting of WT1 in the samples in **(E)** and the quantification shown on the right. The data represent the mean ± SD of three independent experiments. *p < 0.05, statistically significant; **p < 0.01 and ***p < 0.001, statistically very significant.

**Figure 6:**
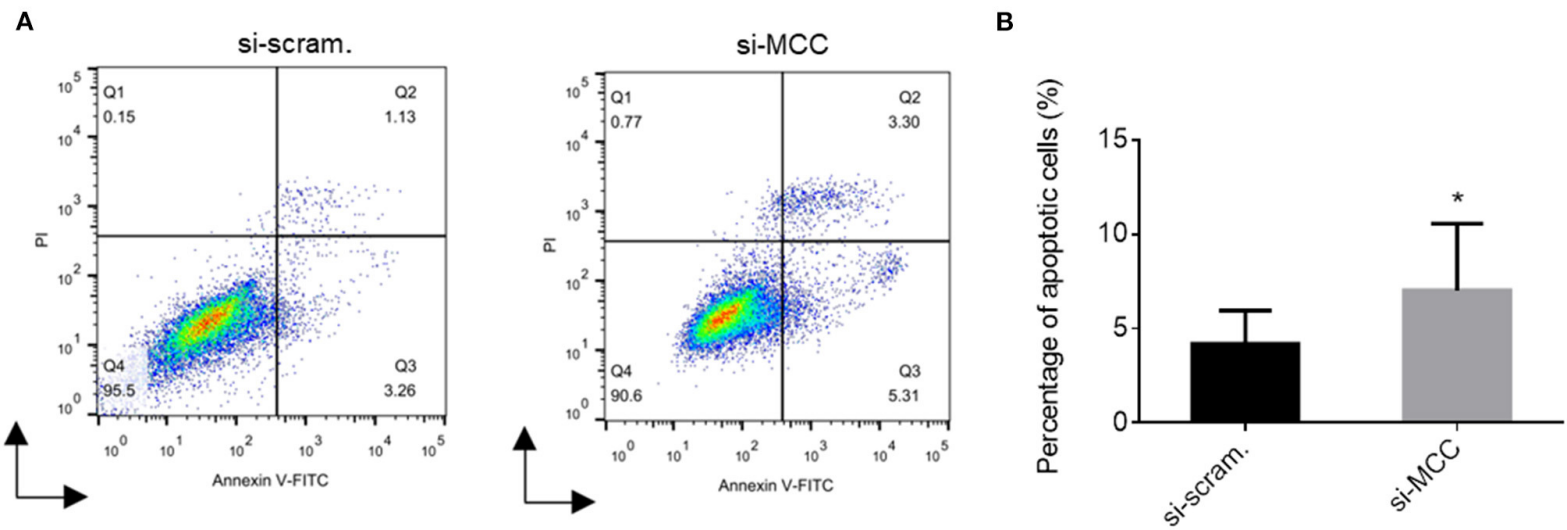
Flow cytometry of Annexin V staining of podocytes treated with scramble control and si-MCC for 72h. **(A)** Histogram representation of the results of the flow cytometric assay. **(B)** Quantification of the percentages of the Annexin V positive cells in the samples. The results were obtained from three independent experiments (*n* = 3) and calculated as mean ± SD. *p < 0.05, statistically significant.

We observed that the podocytes transfected with si-MCC became elongated in morphology 72 h after transfection (Figure 4). In phalloidin staining, si-MCC-treated cells exhibited marked cytoskeletal change with a significant reduction of F-actin stress fibers in the cells (Figures 5A,B). Reduction of actin stress fibers is a sensitive indicator of podocyte injury and is commonly used for assessing the injurious effect of a variety of treatments (20, 21). We next examined the expression of CD2AP and WT1, two podocyte essential genes whose downregulation is usually observed in various injuries, and found that they were downregulated at both mRNA and protein levels (Figures 5C–F). In the flow cytometry of Annexin V staining, si-MCC treated cells exhibited a significantly higher level of apoptosis than the control cells (Figure 6).

### MCC Knockdown Had Limited Effects on Cell Cycle, Wnt/β-Catenin, AKT and ERK in Podocytes

Since MCC has been shown to regulate cell cycle positively or negatively as tumor suppressor gene or oncogene depending on cell types, we examined the expression of cell cycle related genes in podocytes treated with si-MCC by qPCR. Among CDK inhibitors, p21Cip1, p27Kip1 and p57Kip2, but not p15INK4b and p16INK4a, were slightly but significantly downregulated in the podocytes treated with si-MCC for 72 h (Supplementary Figures 1A–E). At the earlier time point of 36 h, p27Kip1 but not the others had already exhibited little reduction. We then examined the expression of other cell cycle genes and found downregulation of CDK1, CDK4, Cyclin B1, Cyclin D2, Cyclin E1 and Cyclin E2 in the cells at 72 h post-transfection of si-MCC (Supplementary Figures 2A,B). At 36 h post-transfection only cyclin E1 and E2 had started to be downregulated while the others remained unchanged. Consistently, downregulation of PCNA was also found in the cells at 72 h but not 36 h post-transfection (Supplementary Figure 2C). We then performed immunoblotting to examine the proteins of p16, p21, p27, p57, CDK1, Cyclin E1, and Cyclin D2, whose antibody was available in the lab. However, the protein levels of these genes were essentially not changed except for p57 and cyclin D2 (Supplementary Figure 3). We also examined PCNA, the marker of cell proliferation, which is upregulated in the transition of G1-S phase, and found that it was not changed. These results together suggested that MCC deficiency had little effect on cell cycle of podocytes.

Since MCC is known to regulate Wnt/β-catenin signaling and even reduces β-catenin expression level (18), we then examined β-catenin in the control and si-MCC cells, but no any difference was found at both 36 and 72 h post siRNA transfection (Supplementary Figures 4A,B). AKT and ERK activities were also examined and compared between si-MCC and si-scramble control cells. Slight reduction of both total AKT and p-AKT was found in the cells at 72 h but not 36 h post siRNA transfection; and no any change was found in ERK and p-ERK levels at any time point (Supplementary Figures 5A–D).

### RNA-Sequencing and Gene set Enrichment Analysis (GSEA) of Genes Regulated in MCC Knockdown Podocytes

**Figure 7:**
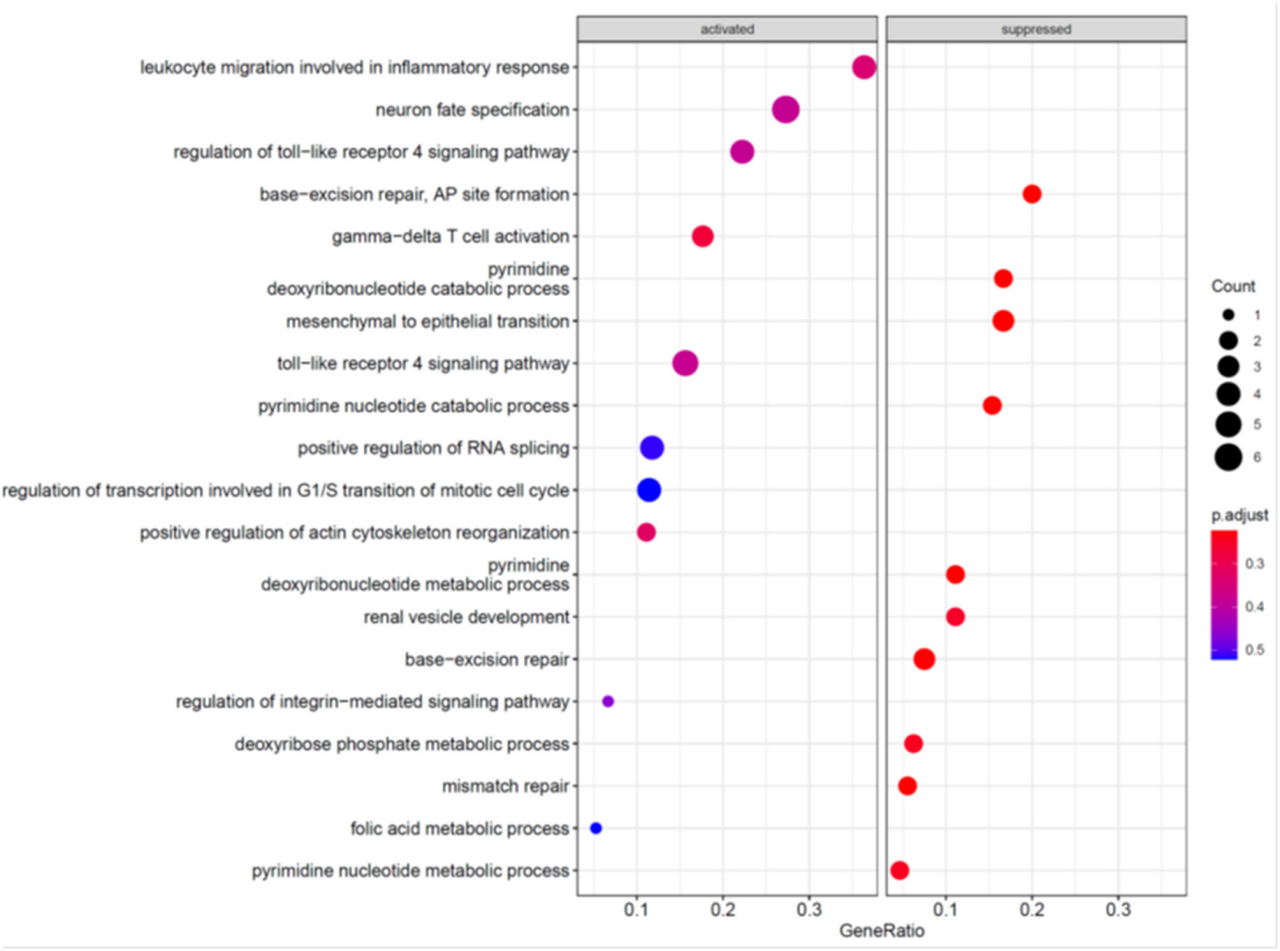
GO_BP analyses of genes regulated in the podocytes deficient in MCC.

**Figure 8:**
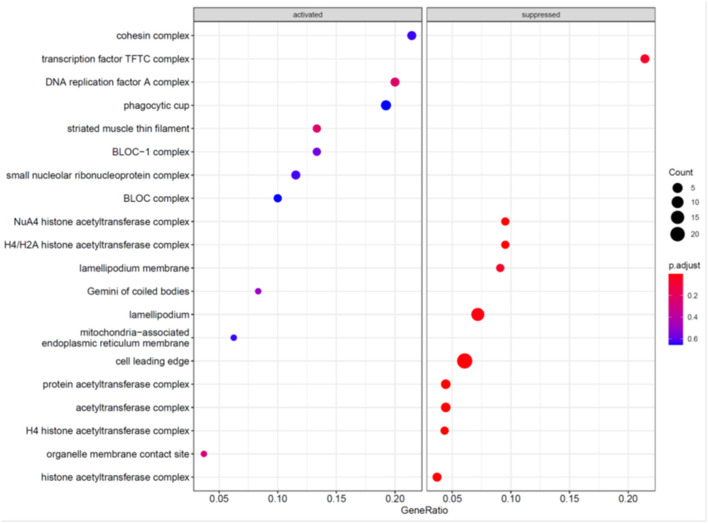
GO_CC analyses of genes regulated in the podocytes deficient in MCC.

To further explore the mechanism underlying the effects of MCC knockdown on podocytes, we performed RNA-seq analysis of MCC knockdown podocytes 24 h post siRNA transfection when MCC mRNA was mostly eliminated in the cells and the cells did not exhibit any overt morphological change (Figure 3). Although the GSEA (GO_BP) showed the activation of “regulation of transcription involved in G1/S transition of mitotic cell cycle”, the enrichment did not reach a statistical significance (Figure 7). We also found activation of inflammatory and immune responses (including TLR-4 signaling, T-cell activation, etc). On the other hand, several enrichments were significantly suppressed, including base-excision/mismatch repair, and mesenchymal to epithelial transition (MET). In the GO_MF analysis, many activities involve nuclei (Supplementary Figure 6), and this is consistent with nucleic localization of MCC as shown in Figure 2 and suggestive of a role for MCC in gene expression regulation. In the GO_CC analysis (Figure 8), we found “lamellipodia membrane”, “lamellipodium” and “cell leading edge”, suggesting a role of MCC in lamellipodia formation of podocytes. KEGG and Reactome analyses were consistent with the GO analysis (Supplementary Figures 7, 8).

### MCC Deficiency Disrupted Lamellipodia Formation

**Figure 9:**
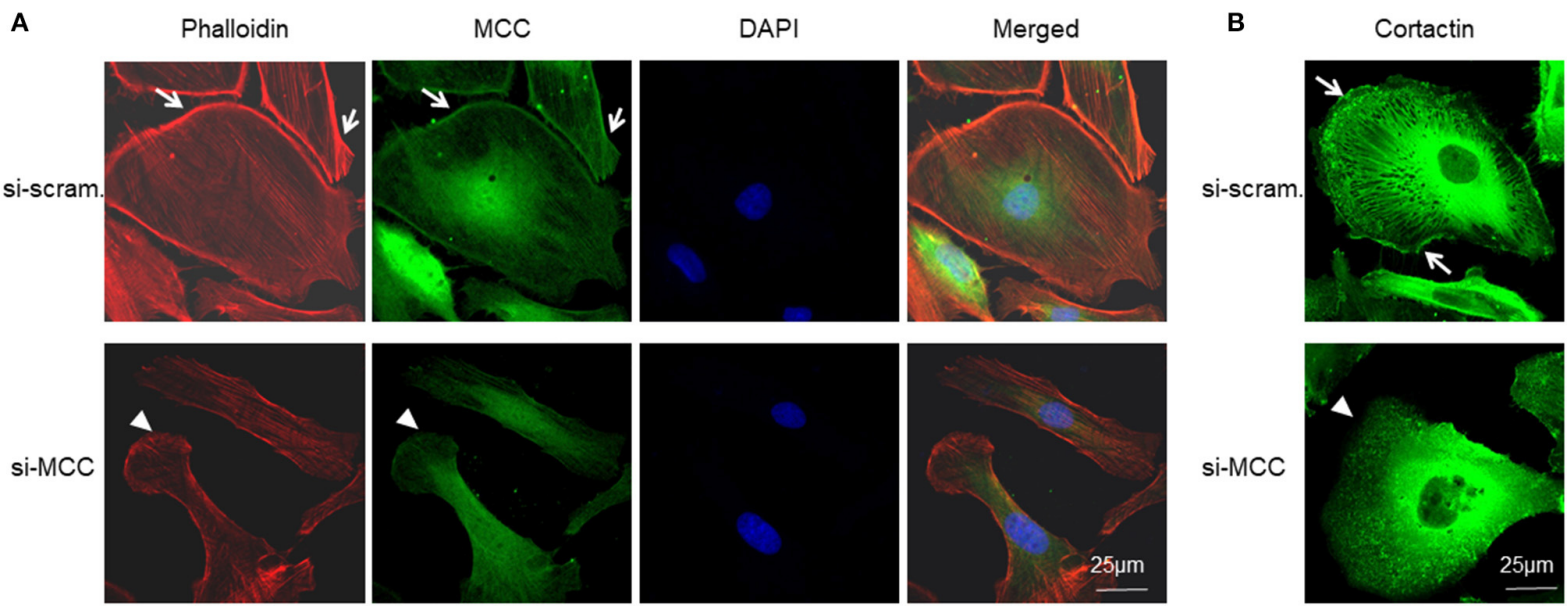
Requirement of MCC in lamellipodium formation of podocytes. **(A)** Fluorescence immunostaining of MCC and F-actin (phalloidin) shows that MCC protein is abundant in lamellipodia (arrows), and its knockdown caused loss of lamellipodia (arrow head) in the podocytes. **(B)** Fluorescence immunostaining of lamellipodial marker, cortactin, further shows the loss of lamellipodia in the cells lacking MCC (arrow head).

To determine the association of MCC with podocyte lamellipodium as revealed by RNA-seq and bioinformatics analysis above, we co-stained MCC with lamellipodium marker, phalloidin-stained F-actin, and observed that MCC was co-localized with F-actin at the periphery of podocytes, indicating that MCC is localized in lamellipodia (Figure 9). In the si-MCC treated podocytes, we were surprised to find that the cells lost most of the lamellipodia as shown by negative staining of phalloidin at the cell periphery (Figure 9A), indicating that MCC is involved in the formation of lamellipodium in podocytes. We examined the integrity of lamellipodia using another marker of lamellipodia, cortactin, which showed presence of lamellipodia in control cells but absence in the podocytes treated with si-MCC (Figure 9B).

### MCC is Downregulated in Injurious Podocytes and Glomerular Diseases

**Figure 10:**
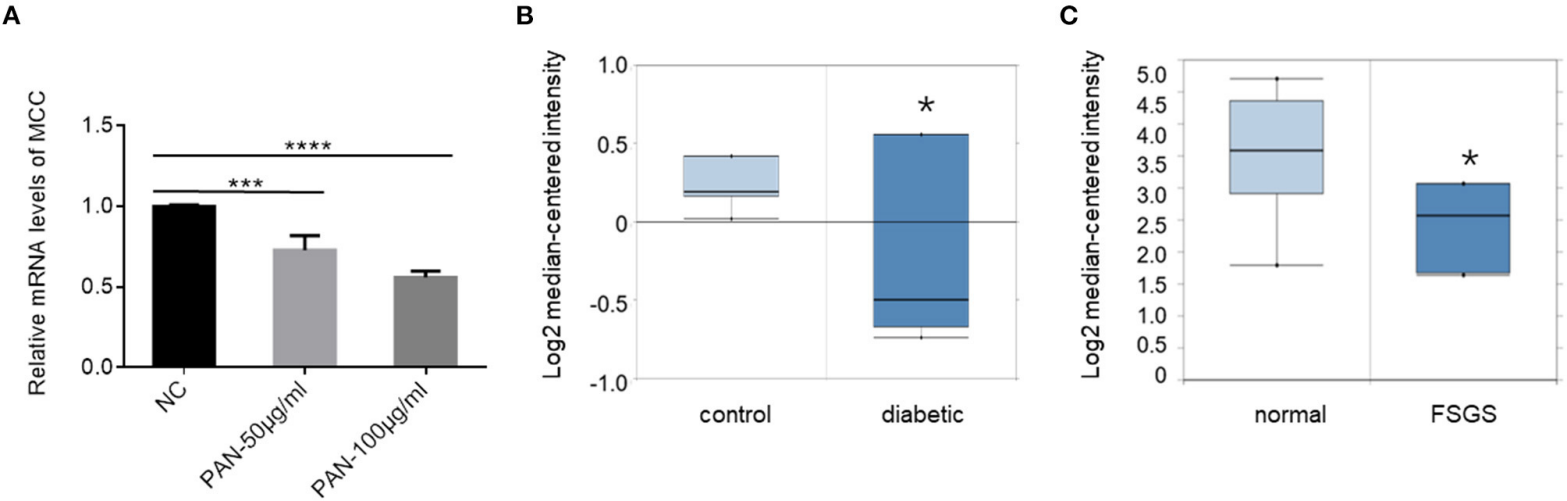
MCC downregulation in podocyte injury and podocytopathy. **(A)** qPCR analysis of cultured podocytes treated with or without PAN for 24 h, showing a significant downregulation of MCC by PAN. The results represent the mean ± SD of three independent experiments; ***p < 0.001, ****p < 0.0001, statistically very significant; **(B)** Nephroseq database search showed MCC downregulation in glomeruli of diabetic mice (eNOS-deficient C57BLKS db/db) (*n* = 7) compared with the non-diabetic control mice (*n* = 5). *p < 0.01; **(C)** Nephroseq database search revealed downregulation of MCC in glomeruli of FSGS patients. Normal control, *n* = 9; FSGS patients, *n* = 6; *p < 0.05, statistically significant.

To explore the role of MCC expression changes in podocytopathy, we first explored whether MCC expression could be affected by injurious stimuli. In cultured podocytes treated with puromycin aminonucleosides (PAN), a commonly used podocyte injury model, we found a significant dose- dependent downregulation of MCC in the cells as assessed by qPCR (Figure 10A). Next, we searched Nephroseq database and found that MCC is significantly downregulated in glomeruli of diabetic mice (Figure 10B), as well as the patients with focal segmental glomerulosclerosis (FSGS) (Figure 10C). These results suggest that MCC downregulation may facilitate podocyte injury in the glomerular diseases.

## Discussion

In the present study, we pursued a previous finding that MCC expression was detected in majority of podocytes that underwent single-cell RNA-sequencing and speculated that MCC might be indispensable for podocytes. Further database mining confirmed MCC expression in mouse podocytes, as well as in human podocytes, supporting a role for MCC in podocytes. We performed siRNA silencing of MCC in cultured podocytes and observed cell injury. Thus, we have identified MCC as a novel podocyte essential gene. We further investigated the potential mechanism underlying the role of MCC and found that it is required for lamellipodia formation in podocytes.

Single-cell RNA-seq is a powerful tool to dissect gene expression in single cells, providing many new insights into the biology and pathology of a given cell type. We previously performed single-cell RNA-seq of mouse podocytes and mesangial cells and identified a number of novel podocyte and mesangial essential genes (20, 29). Our approach for essential genes identification for a cell type is based on the speculation that genes expressed in all individual cells are likely indispensable for the cell type while genes expressed only in a proportion of cells could be dispensable. We performed ultra-deep sequencing of mouse podocytes and identified 335 genes whose expression was detected by RNA-seq at levels >0.5 RPKM. Among the 335 genes, 92 showed > 5-fold expression levels of that in mesangial and endothelial cells and considered to be podocyte specific essential genes. Among the 92 genes, about 30 were already known to be essential for podocytes, thus validating the approach. We took several other approaches to demonstrate the essentiality of the novel candidates (20). In addition to the genes expressed in all sequenced podocytes, there were many other genes whose expression was detected in majority of the cells and we supposed that these genes might be also essential for podocytes and their absence might have arisen from technical variations in the single-cell RNA-seq process. We examined the list of such genes and became interested in MCC which had been shown to act a tumor suppressor and prevent cell cycle progression.

Firstly, we analyzed phenotypes of the podocytes treated with MCC siRNA, including actin stress fiber formation, cell morphology, apoptosis, and the molecules in the injurious pathways. We found that MCC knockdown indeed caused podocyte injury as demonstrated in above assays. We then explored the mechanism underlying the role of MCC in podocytes.

Podocytes are characterized by their terminally differentiated property. Dependent of their nature, injurious stimuli are capable of positively or negatively regulating expression of genes involved in cell cycling, e.g., CDK inhibitor, p21, p27, and p57 (16, 19, 30), resulting in either cell cycle arrest or re-entry. Alteration of p21, p27, and p57 levels was observed in multiple podocyte injury models, including puromycin nephrosis, TGF-β and Angiotensin II, and facilitated podocyte apoptosis in the models (30). In the present study, MCC knockdown resulted in downregulation of p21, p27, and p57 at the mRNA level in the podocytes. However, at the protein level only p57 showed a slight reduction. Downregulation of cell cycle molecules were also observed at the mRNA level, but only cyclin D2 was reduced markedly at the protein level. Regardless of these changes, the cell cycle status of the cells deficient in MCC was maintained as evident by the unchange of PCNA protein, the marker of S-G1 phase transition. Therefore, the role for MCC in podocytes might not involve cell cycle regulation, at least, under normal condition as shown in the present study. It would be interesting to test whether MCC has a role in cell cycle regulation of podocytes under stresses or in diseases.

MCC has been shown to inhibit Wnt/β-catenin signaling (17) and could even directly reduce β-catenin level (18). In our present study with cultured podocytes, we did not observe the change of β-catenin protein level and activity in the podocytes treated with si-MCC. It appears that regulatory activity of MCC in Wnt/β-catenin signaling is also cell type dependent. However, it is necessary to examine β-catenin transcriptional activity in the podocytes to definitely conclude it. In addition, it could be more important to determine whether MCC is involved in β-catenin signaling in podocytes treated with injurious factors.

In the RNA-seq and GSEA analysis, we identified several earliest enrichments in the podocytes upon the elimination of MCC in the cells 24 h post siRNA transfection when there was no any apparent change that could be seen. Interestingly, both cell cycle and Wnt/β-catenin signaling were not significantly enriched, consistent with our experimental results. However, we found that MCC deficiency was associated with “lamellipodia”, “lamellipodia membrane” and “cell leading edge”. We then carefully investigated the issue and found that MCC protein was abundant in the lamellipodia and MCC knockdown led to loss of lamellipodia in the podocytes as shown by the absence of both F-actin and cortactin at the periphery of the cells. *In vivo*, we performed MCC and SYNPO co-staining with kidney and found that MCC co-localized along GBM, indicating that MCC is present in foot processes of podocytes (Figure 1G). Foot process localization of MCC *in vivo* is consistent with its localization in lamellipodia of cultured podocytes. Lamellipodia is essential for the foot process and slit diaphragm formation of podocytes (31, 32). Therefore, MCC may be also involved in these processes thereby underlying its essentiality for podocytes. In fact, involvement of MCC in lamellipodia formation has been reported in colon epithelial cells, and mechanistically, MCC binds to Scrib and its downstream partner Myosin-IIB in a multiprotein complex (33). Scrib and Myosin-IIB are both highly expressed in podocytes according to HPA and other sources of data (data not shown). It would be interesting to test whether MCC regulates lamellipodia in a similar way.

From a translational perspective, we found that MCC can be downregulated by PAN in podocytes. Furthermore, MCC was found significantly downregulated in glomeruli of diabetic mice and FSGS patients according to Nephroseq database. MCC downregulation may facilitate the process of podocyte injury and disease development. Further investigation of the exact molecular mechanism underlying MCC downregulation-mediated podocyte injury is warranted. There is a pitfall when one claims downregulation of a gene in podocytes based on quantification with glomerular material, e.g., RNA and protein. This is because podocyte loss contributes to the reduction of podocyte gene expression in the glomerular material, likely causing false downregulation of the podocyte gene. To definitely conclude MCC downregulation in podocytes of DKD and FSGS, we used Nephroseq data to compare the fold changes of MCC with that of podocyte marker genes, MAGI2, LMX1B, WT1, FOXC1, FOXC2, VEGFA, SYNPO, NPHS1, NPHS2, YAP1 and CD2AP, which are well known to be downregulated in the same diseases. We found that MCC had a similar reduction to those of the podocyte markers, confirming that MMC is downregulated in the diabetic nephropathy and FSGS (Supplementary Figures 9, 10).

In conclusion, our present study has identified a novel podocyte essential gene, MCC, which plays an essential role in the formation of lamellipodia in the cultured podocytes, and likely in the formation of podocyte foot processes and slit diaphragms in animal. MCC downregulation may facilitate podocyte injury. Therefore, reversal of MCC downregulation in podocytes may be a potential therapeutic approach for podocytopathy. Although the present *in vitro* study has identified MCC as a novel essential gene of podocytes and shown some potential mechanistic insights into its role in lamellipodium formation, further studies, particularly *in vivo*, are required to better understand the function and mechanism of MCC in podocyte pathophysiology.

## Data Availability Statement

The datasets presented in this study can be found in online repositories. The names of the repository/repositories and accession number(s) can be found below: https://www.ncbi.nlm.nih.gov/geo/, GSE186547.

## Author Contributions

SS conceived the study. HS, ZL, WH, and SS designed the experiments. HS, LZ, and XX performed the experiments. JS performed bioinformatics analysis. HS, SS, and ZL interpreted data and wrote the manuscript. All authors have reviewed and approved the final version of the manuscript.

## Funding

This work was supported by the grant supports from the National Natural Science Foundation of China (81970619 and 81770701), the Social Development Project of Jiangsu Province (BE2020698), and the National Key Clinical Programs for Army – Nephrology Project (2014ZDZK001).

## Conflict of Interest

The authors declare that the research was conducted in the absence of any commercial or financial relationships that could be construed as a potential conflict of interest.

## Publisher's Note

All claims expressed in this article are solely those of the authors and do not necessarily represent those of their affiliated organizations, or those of the publisher, the editors and the reviewers. Any product that may be evaluated in this article, or claim that may be made by its manufacturer, is not guaranteed or endorsed by the publisher.
