# Supplementary material for: MCC Regulator of WNT Signaling Pathway (MCC) Is a Podocyte Essential Gene
Source: Front Med (Lausanne). 2021 Dec 2;8:777563. doi: 10.3389/fmed.2021.777563 (PMC8674659; doi:10.3389/fmed.2021.777563)
Supplement: Supplementary file 1 [file Data_Sheet_1.pdf]

Supplementary Information

MCC regulator of WNT signaling pathway (MCC) is a podocyte essential gene

Hui Song, Lulu Zhuang, Xiaodong Xu, Jingsong Shi, Weixin Hu, Zhihong Liu, Shaolin Shi

Supplemental Figures

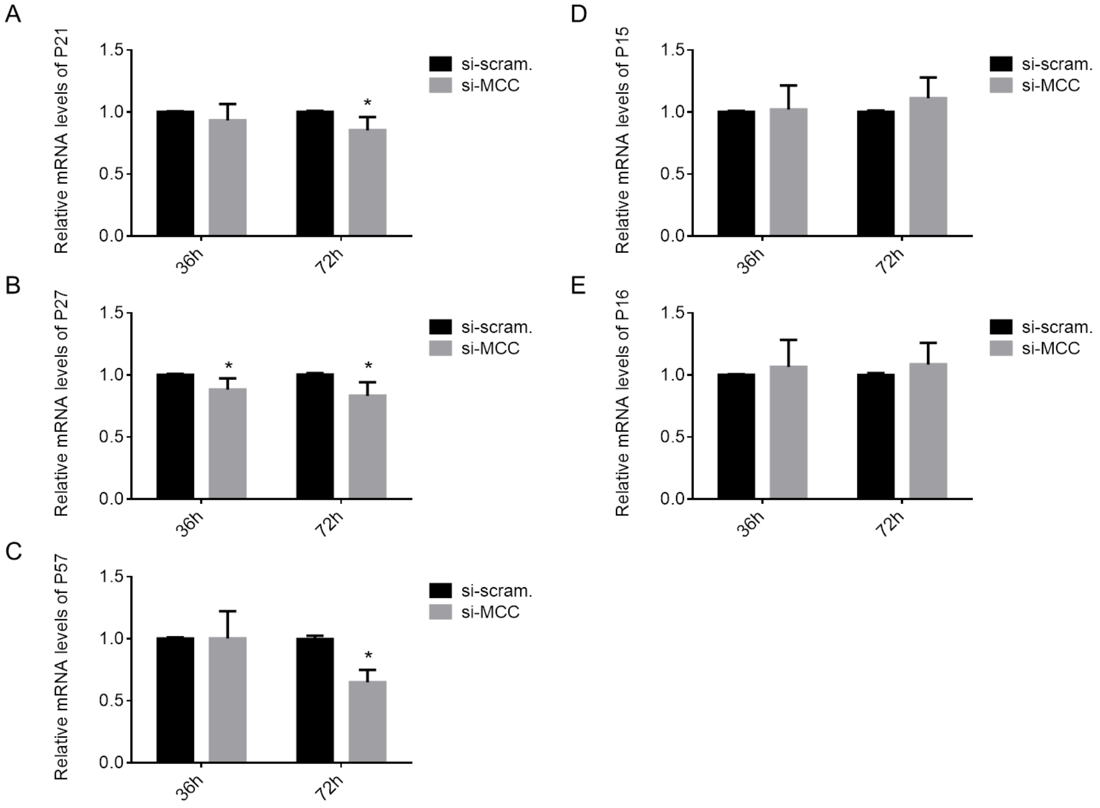

Supplemental Figure 1. The expression of CDK inhibitors in podocytes with MCC knockdown. qPCR analysis of CDK inhibitors in the cells treated with scramble control and si-MCC at 36 h and 72 h post transfection of siRNA. A. p21Cip1; B. p27Kip1; C. p57Kip2; D. p15INK4b; E. p16INK4a. Each result represents the mean±SD of three independent experiments. *$p<0.05$, statistically significant.

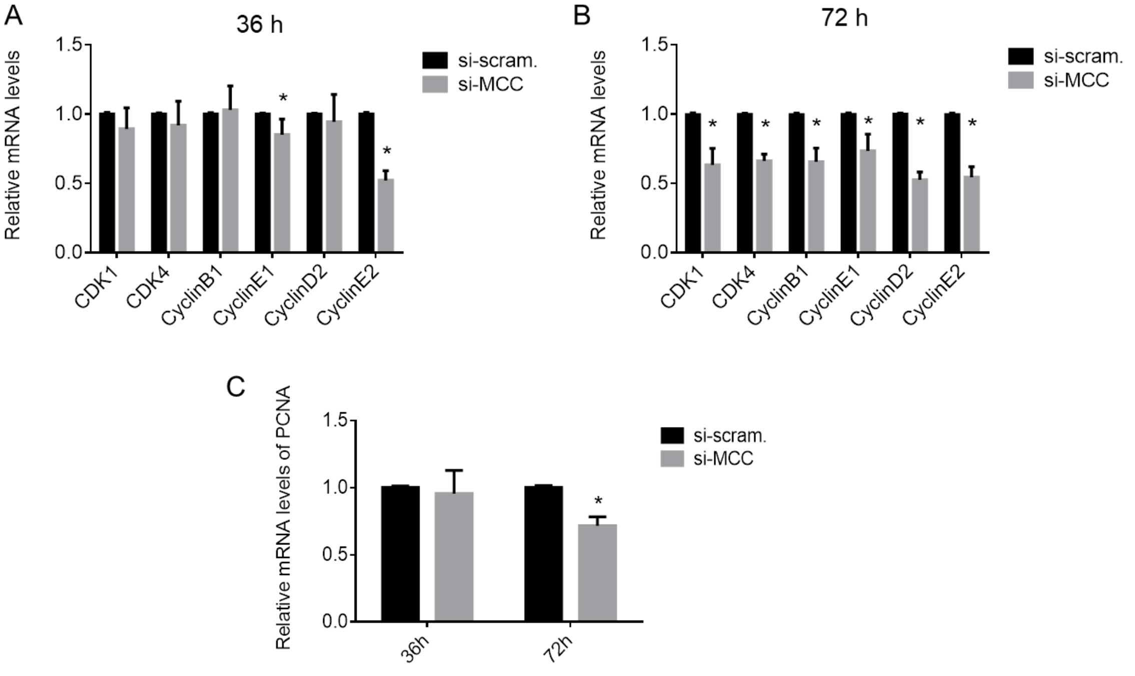

Supplemental Figure 2. MCC knockdown resulted in downregulation of expression of cell cycle related genes. A, B. qPCR analysis of the cell cycle regulatory genes in the cells treated with scramble control and si-MCC for 36 h (A) and 72 h (B), respectively. B. qPCR analysis of PCNA in the same cells as in A and B. The results were from three independent experiments and calculated as mean ± SD. *p<0.05 denotes statistically significant.

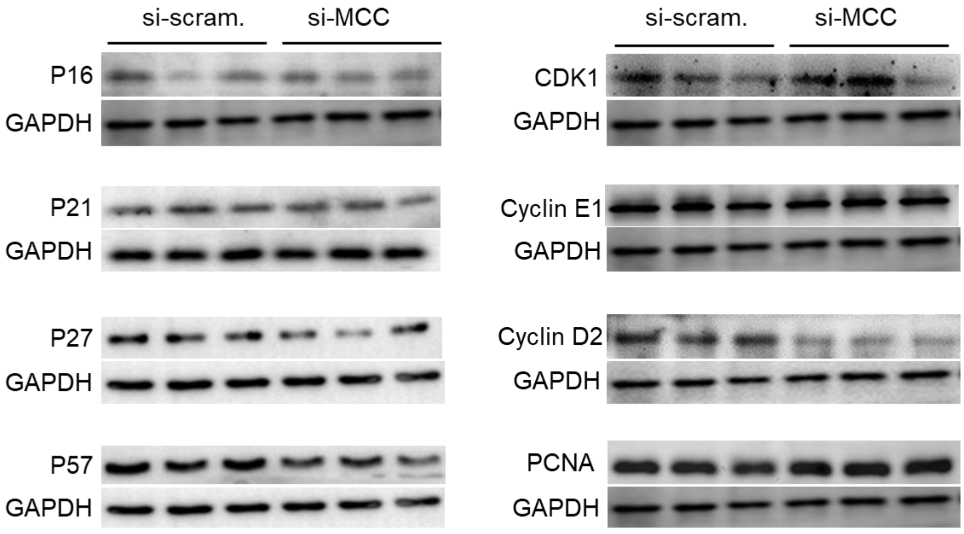

Supplemental Figure 3. The protein levels of cell cycle genes in podocytes treated with scramble control and si-MCC, respectively. Immunoblotting of these proteins revealed reduction of p57 and cyclin D2 in the si-MCC treated cells, while the others were not changed.

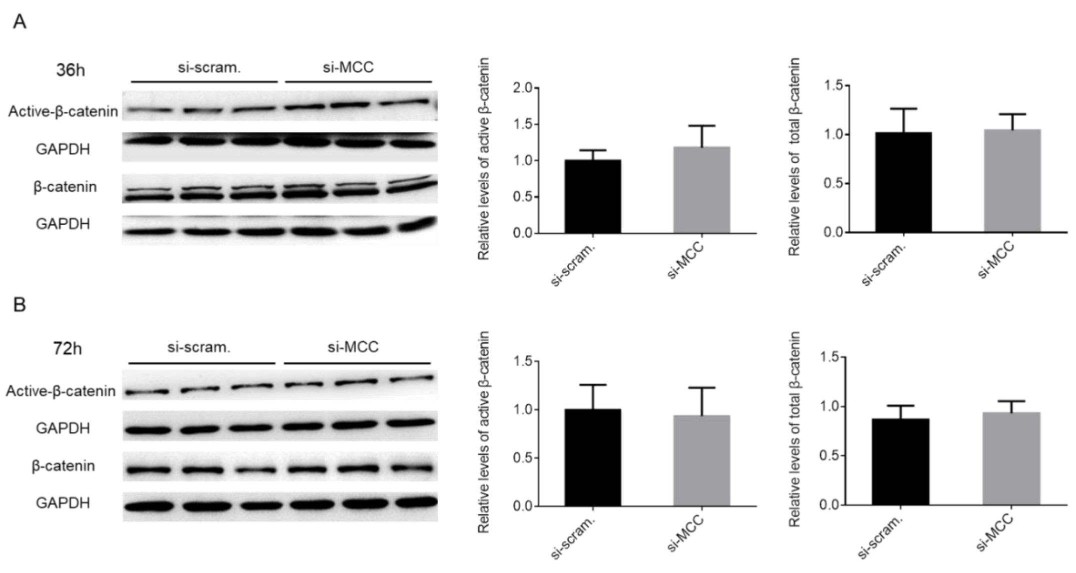

Supplemental Figure 4. MCC deficiency did not affect the level and activity of β-catenin. A, B. Immunoblotting of phosphorylated and total β-catenin in podocytes treated with scramble control and si-MCC, respectively, for 36 h (A) and 72 h (B). Quantifications of the results were shown on the right. The results were obtained from three independent experiments and calculated as mean ± SD. no any statistically significant difference was found.

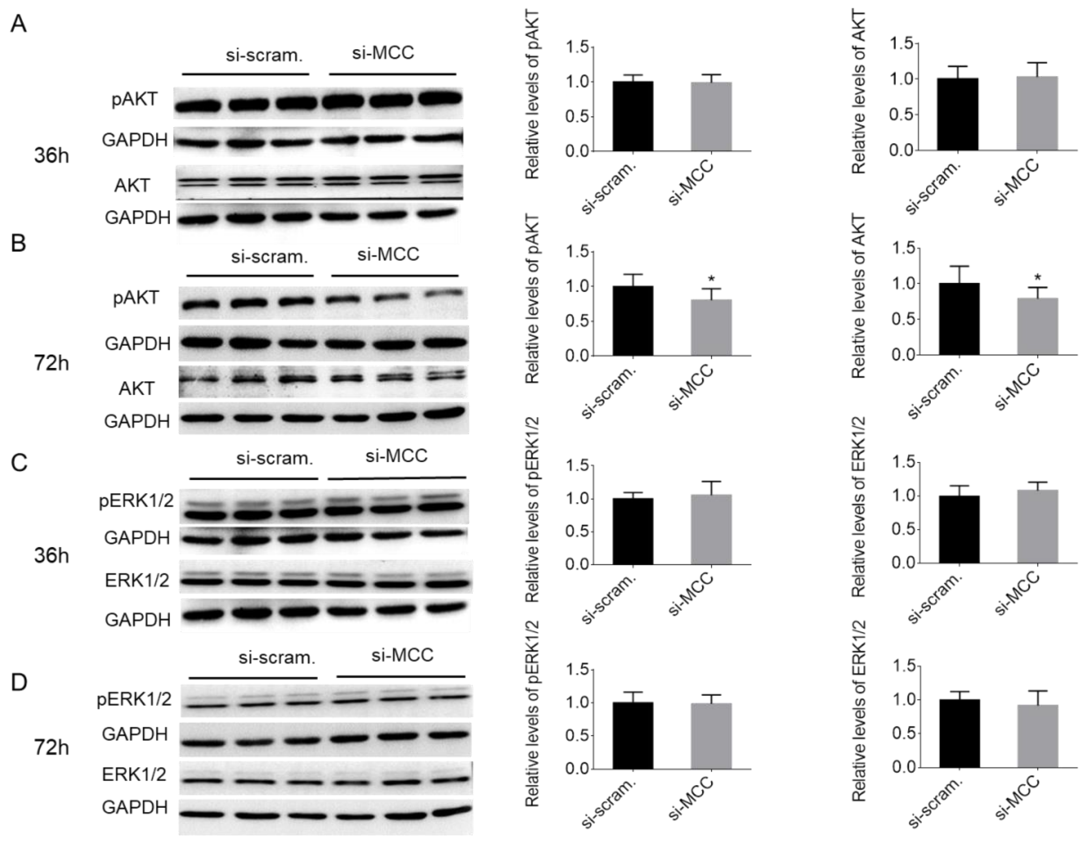

Supplemental Figure 5. MCC knockdown did not affect the level and activity of AKT and ERK. A, B.

Immunoblotting of phosphorylated and total AKT in podocytes treated with scramble and si-MCC for 36 (A) and 72 h (B), respectively. Quantifications of the results were shown on the right. C, D. Immunoblotting and quantification of the phosphorylated and total ERK in the same cells as in A (C) and B (D). The results represent the mean ± SD from three independent experiments. *p<0.05 denotes a difference statistically significant.

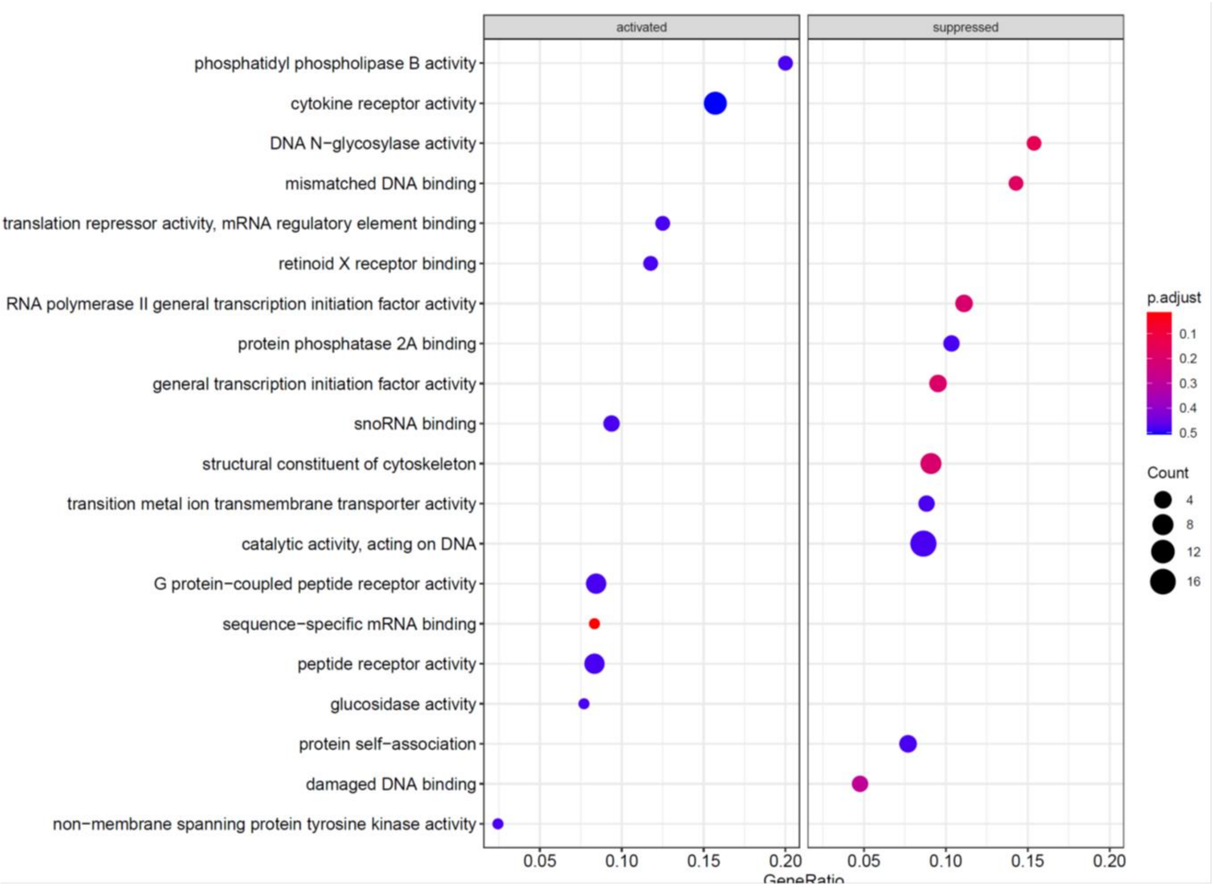

Supplemental Figure 6. GO_MF analysis of genes regulated in the podocytes deficient in MCC.

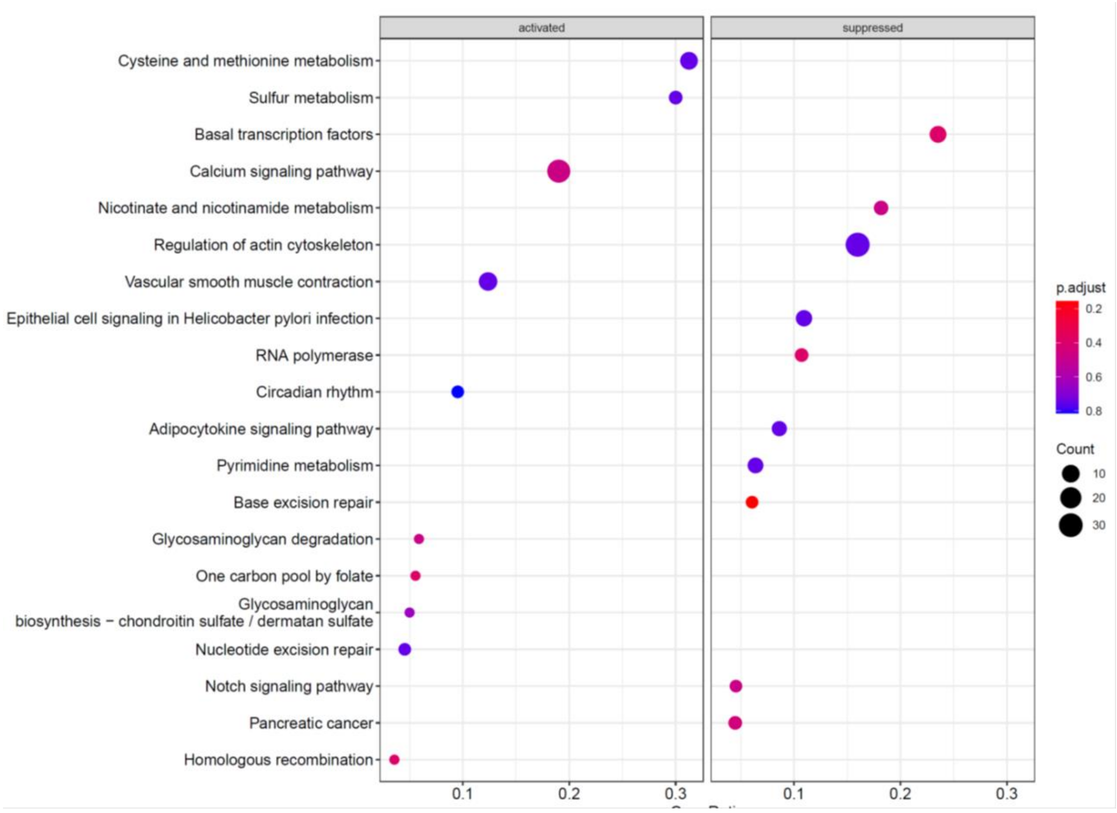

Supplemental Figure 7. KEGG pathway analysis of genes regulated in podocytes deficient in MCC.

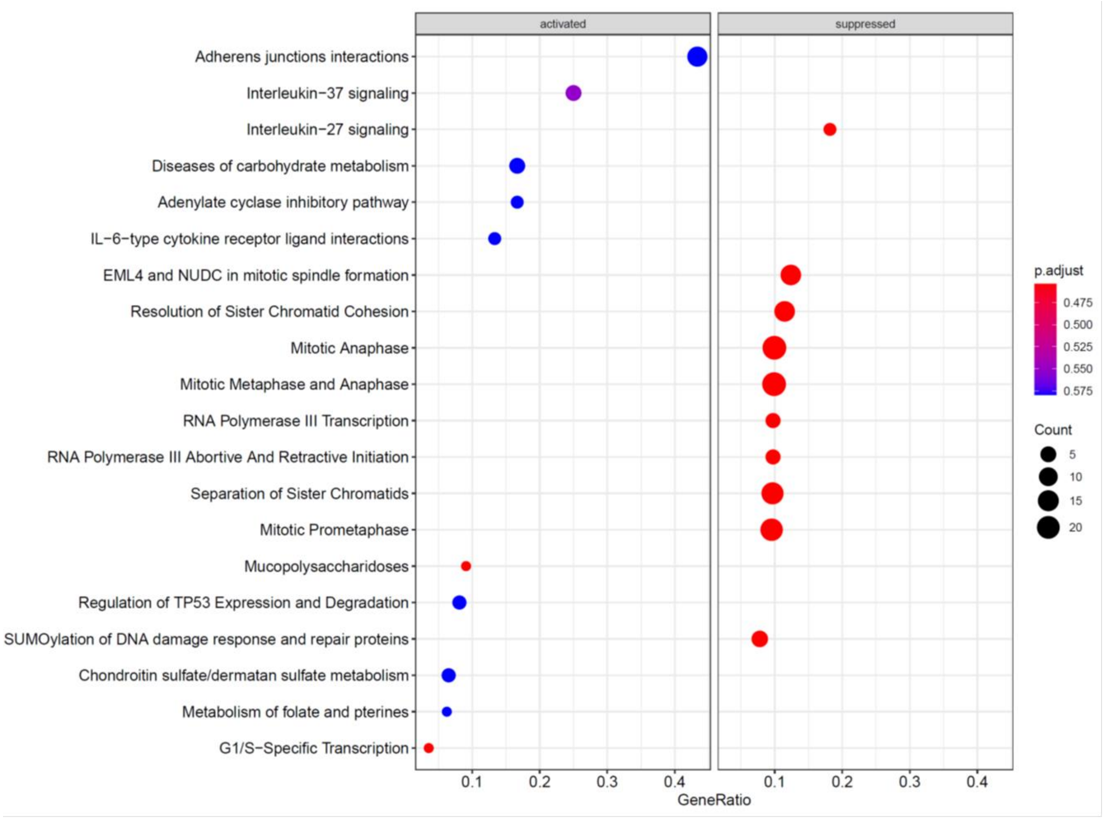

Supplemental Figure 8. GO_Reactome analysis of genes regulated in the podocytes deficient in MCC.

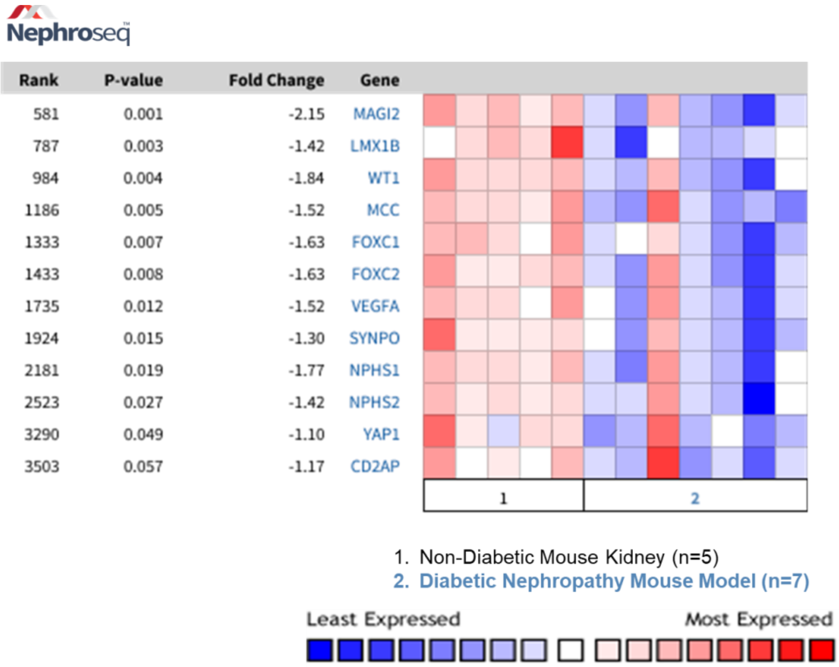

Rank	P-value	Fold Change	Gene
581	0.001	-2.15	MAGI2
787	0.003	-1.42	LMX1B
984	0.004	-1.84	WT1
1186	0.005	-1.52	MCC
1333	0.007	-1.63	FOXC1
1433	0.008	-1.63	FOXC2
1735	0.012	-1.52	VEGFA
1924	0.015	-1.30	SYNPO
2181	0.019	-1.77	NPHS1
2523	0.027	-1.42	NPHS2
3290	0.049	-1.10	YAP1
3503	0.057	-1.17	CD2AP

Supplemental Figure 9. Comparison of selected genes in the dataset of Hodgin Diabetes Mouse Glom (www.nephroseq.org): glomeruli of non-diabetic mice versus eNOS-deficient C57BLKS db/db mice.

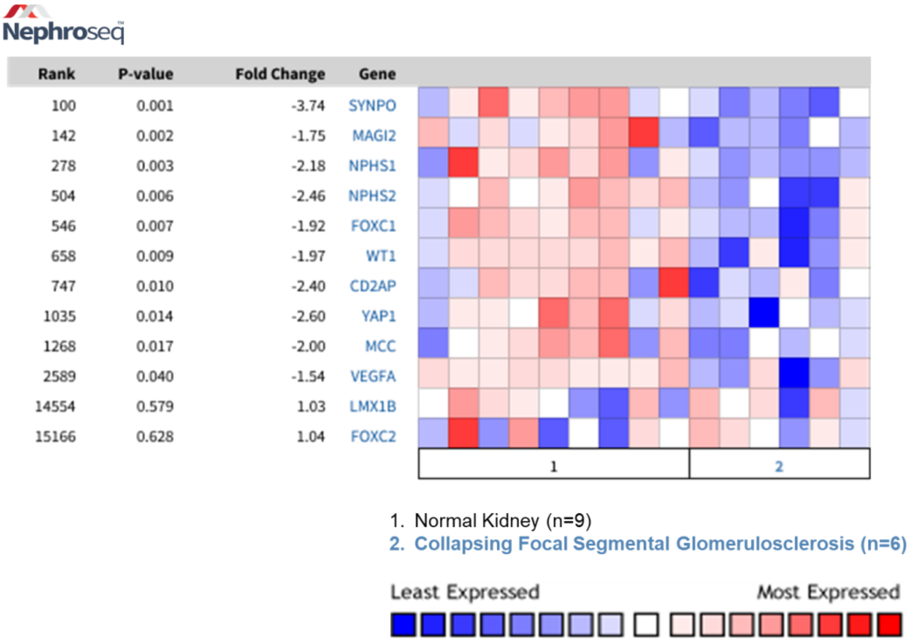

Rank	P-value	Fold Change	Gene
100	0.001	-3.74	SYNPO
142	0.002	-1.75	MAGI2
278	0.003	-2.18	NPHS1
504	0.006	-2.46	NPHS2
546	0.007	-1.92	FOXC1
658	0.009	-1.97	WT1
747	0.010	-2.40	CD2AP
1035	0.014	-2.60	YAP1
1268	0.017	-2.00	MCC
2589	0.040	-1.54	VEGFA
14554	0.579	1.03	LMX1B
15166	0.628	1.04	FOXC2

Supplemental Figure 10. Comparison of selected genes in the dataset of Hodgin FSGS Glom (www.nephroseq.org): glomeruli of normal kidney versus that with collapsing focal segmental glomerulosclerosis.

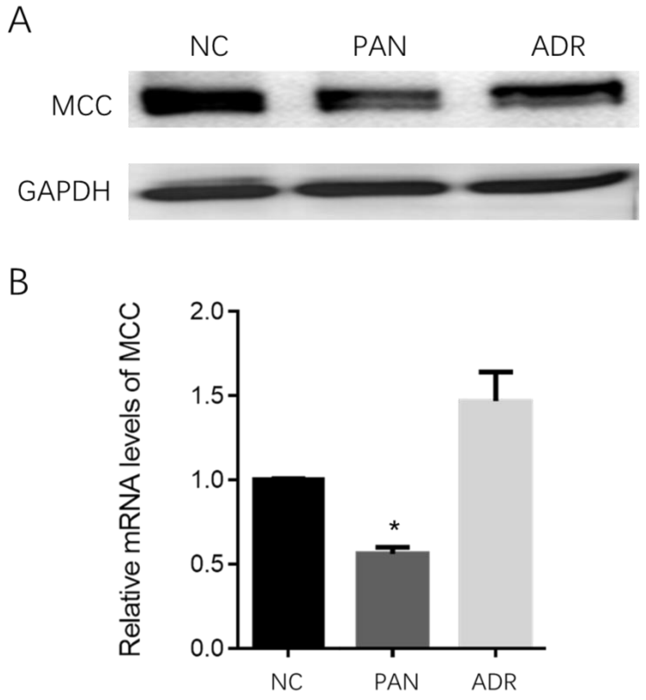

Supplemental Figure 11. MCC was downregulated by PAN but not ADR in cultured podocytes. A. Representative western blotting showing downregulation of MCC by PAN but not ADR in podocytes. B. qPCR analysis showed downregulation of MCC by PAN but not ADR in the cells. The results represent the mean ± SD from three independent experiments (n=3). *p<0.05 denotes a difference statistically significant.
